# Population Pharmacokinetics of Isavuconazole in Subjects with Mild or Moderate Hepatic Impairment

**DOI:** 10.1128/AAC.02942-15

**Published:** 2016-04-22

**Authors:** Amit Desai, Anne-Hortense Schmitt-Hoffmann, Salim Mujais, Robert Townsend

**Affiliations:** a Astellas Pharma Global Development, Inc., Northbrook, Illinois, USA; b Basilea Pharmaceutica International Ltd., Basel, Switzerland

## Abstract

Isavuconazole, administered as the prodrug isavuconazonium sulfate, was recently approved by the U.S. Food and Drug Administration and the European Medicines Agency for the treatment of adults with invasive aspergillosis and mucormycosis. The objective of this analysis was to develop a population pharmacokinetic model using NONMEM (version 7.2) for subjects with hepatic impairment, using intravenous and oral administration data from two hepatic studies, and to simulate concentration profiles to steady state, thus evaluating the need for dose adjustment. A two-compartment model with Weibull absorption function and first-order elimination process adequately described plasma isavuconazole concentrations. The population mean clearance in healthy subjects was 2.5 liters/h (5th and 95th percentiles: 2.0 and 3.1). The mean clearance values for subjects with mild and moderate hepatic impairment decreased approximately to 1.55 liters/h (5th and 95th percentiles: 1.3 and 1.8 liters/h) and 1.32 liters/h (5th and 95th percentiles: 1.05 and 1.35), respectively. Peripheral volume of distribution increased with body mass index. Simulations of mean concentration time profiles to steady state showed less than a 2-fold increase in mean trough concentrations for subjects with mild and moderate hepatic impairment compared with healthy subjects. After administration of the single dose, safety data for subjects with mild and moderate hepatic impairment were generally comparable to those for healthy subjects in both studies. Due to the <2-fold increase in trough concentrations and the established safety margin, dose adjustment appears to be unnecessary in subjects with mild or moderate hepatic impairment.

## INTRODUCTION

Despite the discovery of new antifungal agents within the past decade, invasive fungal infections remain a substantial cause of morbidity and mortality in immunocompromised individuals (1, 2). Isavuconazole, administered as the prodrug isavuconazonium sulfate, is a novel, broad-spectrum triazole antifungal agent developed for the treatment of invasive fungal infections. Recently, isavuconazonium sulfate has been approved by the U.S. Food and Drug Administration (FDA) for the treatment of adults with invasive aspergillosis and invasive mucormycosis (3) and by the European Medicines Agency for the treatment of adults with invasive aspergillosis and those with mucormycosis for whom amphotericin B is inappropriate (4).

Triazole antifungal agents are an important treatment option, but these drugs act as inhibitors of hepatic cytochrome P450 enzymes and most are metabolized by the liver (5), thereby requiring consideration of liver function in treated patients. Hepatic impairment can result in marked changes in the pharmacokinetic (PK) profile of a drug (6). For example, caution is recommended when administering fluconazole to patients with hepatic dysfunction (7), and dose adjustment of voriconazole is recommended for patients with mild to moderate hepatic cirrhosis (8). Increases in posaconazole exposure have also been observed in subjects with hepatic impairment, although no dose adjustment is recommended for those patients (9). As per guidance from the FDA, PK studies in a population with hepatic impairment are important to help in understanding the initial dosing guidance for patients with liver impairment (10).

Isavuconazole is predominantly excreted in feces in bile duct-cannulated rats (A. Schmitt-Hoffmann and W. F. Richter, presented at the 22nd European Congress of Clinical Microbiology and Infectious Diseases, London, United Kingdom, 31 April to 3 March 2012). In humans, isavuconazole is predominantly metabolized; following a single oral dose of cyano-^14^C-labeled prodrug isavuconazonium sulfate, approximately 46.1% of the total sample radioactivity was recovered in feces after 600 h (Astellas Pharma US, Inc., unpublished data). Renal excretion of unchanged isavuconazole accounted for less than 1% of the dose administered (11). Since metabolism is the main route of elimination, with an additional contribution of biliary excretion, the study of hepatic impairment is highly relevant, as isavuconazole PK might be affected by liver disease.

Two studies have been conducted to evaluate the PK of isavuconazole in subjects with hepatic impairment following oral and intravenous administration. The first study assessed subjects with hepatic impairment caused by alcoholic cirrhosis and was reported previously (12). In the current population pharmacokinetic (PPK) analysis, the PK data obtained from that study were combined with PK data for subjects with mild and moderate hepatic impairment caused by chronic hepatitis B and/or C (A. Schmitt-Hoffmann, J. Spickermann, P. Thomann, and B. Roos, presented at the 49th Interscience Conference on Antimicrobial Agents and Chemotherapy, San Francisco, CA, 12 to 15 September 2009). The primary objectives of this analysis were to develop a combined PPK model for subjects with hepatic impairment, to simulate the concentration-time profile for a population with mild and moderate hepatic impairment compared with healthy subjects, and to evaluate the necessity of a possible dose adjustment in patients with hepatic impairment.

## MATERIALS AND METHODS

### Studies.

Data from two phase 1 studies were used to develop the PPK model. Both studies were single-dose parallel studies in which subjects randomly received isavuconazole administered as prodrug isavuconazonium sulfate either orally (p.o.) or by intravenous (i.v.) infusion. All subjects provided written informed consent. A brief summary of the data used for modeling is provided in Table 1.

**TABLE 1:** Description of data used for modeling^*a*^

Study	Design and objective	Treatment	Route	No. of subjects	Population	Pharmacokinetic sampling times
1	Single-dose, randomized, parallel group study	100 mg	p.o./i.v.	48	Healthy subjects, subjects with mild and moderate hepatic impairment due to alcoholic cirrhosis (male and female)	Predose to 480 h postdose
2	Single-dose, randomized, parallel group study	100 mg	p.o./i.v.	48	Healthy subjects, subjects with mild and moderate hepatic impairment due to hepatitis B or C (male and female)	Predose to 480 h postdose

a i.v., intravenous administration; p.o., oral administration. Treatment consisted of isavuconazonium corresponding to 100 mg of isavuconazole.

### Subjects.

The subjects enrolled in both studies were healthy male and female subjects or individuals with mild (Child-Pugh class A) or moderate (Child-Pugh class B) liver disease caused either by alcoholic cirrhosis or chronic hepatitis B and/or C. Healthy subjects were matched to subjects with liver disease on the basis of age (within ±7 years), sex, body weight (within ±8 kg), and body mass index (BMI; within ±4 kg/m^2^). A description of subjects' demographics is presented in Table 2.

**TABLE 2:** Demographics and baseline characteristics

Characteristic (baseline)	Value for of subjects^*a*^		
	Healthy (*n* = 32)	With mild hepatic impairment (*n* = 32)	With moderate hepatic impairment (*n* = 32)
Sex, no. (%)			
Male	21 (66)	21 (66)	21 (66)
Female	11 (34)	11 (34)	11 (34)
Smoking status, no. (%)			
Smoker	19 (59)	24 (75)	24 (75)
Nonsmoker	13 (41)	8 (25)	8 (25)
Median age (range), yrs	50 (40–64)	54 (37–64)	54 (42–64)
Median ht (range), cm	171 (148–185)	168 (147–180)	171 (155–180)
Median wt (range), kg	78 (53–107)	76 (56–105)	76 (58–103)
Median body mass index (range), kg/m^2^	27 (22–33)	28 (21–34)	26 (22–34)

a Values are rounded to the nearest decimal.

### Study design.

Healthy matched subjects and subjects with mild or moderate hepatic impairment were randomly assigned to receive a single dose of isavuconazonium sulfate at 186 mg, equivalent to 100 mg of isavuconazole, either p.o. or via i.v. (2 h) continuous infusion; hereafter, only isavuconazole and the dosing equivalent is used. Plasma samples were collected prior to administration of isavuconazole and at 0.5, 1, 2, 3, 4, 6, 8, 10, 24, 48, 72, 96, 120, 144, 168, 216, 288, 360, 432, and 480 h after administration of the drug in both studies.

### Safety assessments.

Samples were collected for assessment of liver and renal function tests. Samples were collected predose and on days 1, 2, 3, 10, 16, and 21 after administration of isavuconazole. The results of a 12-lead electrocardiogram (ECG) were recorded four times on the first study day as well as on study days 2, 3, 4, 10, and 16. Subjects were monitored for adverse events throughout the study.

### Data description.

A total of 2,016 concentrations from 96 subjects were used for modeling (672 concentrations each from healthy subjects, subjects with mild impairment, and subjects with moderate impairment). A summary of study design, drug administration, and blood sampling is presented in Table 1.

### Data analysis.

The population analyses were conducted via nonlinear mixed-effects modeling with the software program NONMEM (version 7.2; GloboMax LLC, Hanover, MD) on a Hewlett-Packard model Z400, 64-bit personal computer, using Pirana (version 2.7.0; http://www.pirana-software.com). The first-order conditional estimation method in NONMEM was employed for all model runs. Model selection was driven by the data and based on various goodness-of-fit criteria, which included visual inspection of diagnostic scatter plots (observed versus individual predicted concentration), successful convergence of the minimization routine with at least two significant digits in parameter estimates; precision of parameter estimates, and the minimum objective function value and number of estimated parameters.

### Structural pharmacokinetic model.

A variety of linear compartmental PK models were explored to describe total isavuconazole concentration-time data. The base PPK model included two and three compartments, with either simple first-order absorption models or the Weibull absorption model. All random effects were treated as log-normally distributed. The ln-ln transformations of both the model and the data were used to stabilize the residual variance. The residual variance was finally modeled as additive in nature. The models were coded using the NONMEM subroutines for prediction of PPK parameters.

### Pharmacokinetic model with covariates.

Following development of the base structural and statistical PPK model, a covariate analysis was conducted and the best covariate model was selected. Stepwise covariate modeling (SCM) was performed in Perl-speaks-NONMEM (psn.sourceforge.net) by forward-inclusion and backward-elimination steps. In the forward-inclusion step, covariates were added to the model one at a time, using a *P* value of 0.01 for entry into the model. In the backward-elimination step, covariates were removed one at a time using a *P* value of 0.001 for retention in the model. The process was continued until all remaining covariates were significant. Table 2 shows the demographic covariates that were analyzed in the model. In addition to covariates listed in Table 2, the liver function index (HEP0, HEP1, and HEP2) for healthy subjects and subjects with mild and moderate hepatic impairment, respectively, was also analyzed.

### Model validation.

For the best covariate model, population and individual PK parameters were estimated and the precision of the population model parameters (e.g., asymptotic standard errors or bootstrap 95% confidence intervals [CIs]) were generated. Nonparametric bootstrapping, using 500 replications, was used to provide validation of the model parameter estimates. Normalized prediction distribution errors (NPDE) were also plotted to evaluate the best model (13).

### Simulations.

Monte Carlo simulations were performed by using population estimates from the best covariate model. A total of 2,000 concentration-time profiles were simulated to steady state for healthy subjects and subjects with mild and moderate hepatic impairment. Demographic covariates, if significant, were randomly added based on values found in the National Health and Nutrition Examination Survey (NHANES; http://www.cdc.gov/nchs/nhanes.htm). Concentration-time profiles were simulated using the clinical dosing regimen of a dose of isavuconazole at 200 mg every 8 h for 2 days, followed by isavuconazole at 200 mg once daily.

## RESULTS

### Population pharmacokinetic analysis: base model.

A total of 96 subjects were part of the data set used for modeling purposes. In cases of hepatic impairment due to alcoholic cirrhosis, Child-Pugh scores averaged 5.18 for subjects with mild hepatic impairment and 7.43 for subjects with moderate hepatic impairment. For hepatic impairment due to hepatitis B and/or C, Child-Pugh scores averaged 5.75 for subjects with mild hepatic impairment and 8.31 for subjects with moderate hepatic impairment. The values for serum bilirubin, albumin and prothrombin time are presented in Table 3. The PK model development process resulted in a base model that included two compartments with Weibull absorption function. The model had interindividual variability on clearance (CL), volume of distribution of central compartment (*V*_1_), intercompartmental CL (Q), and volume of distribution of peripheral compartment (*V*_p_) and on Weibull absorption parameters; RA, KAMAX, and GAM1. Modeling of isavuconazole PPK data then proceeded with an exploratory graphical inspection of potential covariate parameter relationship for the primary covariate of interest.

**TABLE 3:** Child-Pugh classifications^*a*^

Liver disease	Severity	Serum bilirubin (μmol/liter)	Serum albumin (g/dl)	Prothrombin time (s)	Child-Pugh score
Alcoholic cirrhosis	Mild hepatic impairment	21.03 (10.12)	4.46 (0.41)	1.11 (0.16)	5.18 (0.43)
	Moderate hepatic impairment	49.47 (29.02)	3.76 (0.52)	1.28 (0.27)	7.43 (0.72)
Hepatitis B and/or C	Mild hepatic impairment	82.30 (38.43)	4.07 (0.44)	1.56 (0.97)	5.75 (0.44)
	Moderate hepatic impairment	128.18 (39.10)	3.13 (0.37)	3.20 (1.25)	8.31 (0.70)

a Values are means, with standard deviations in parentheses.

### Best model with covariates.

**FIG 1:**
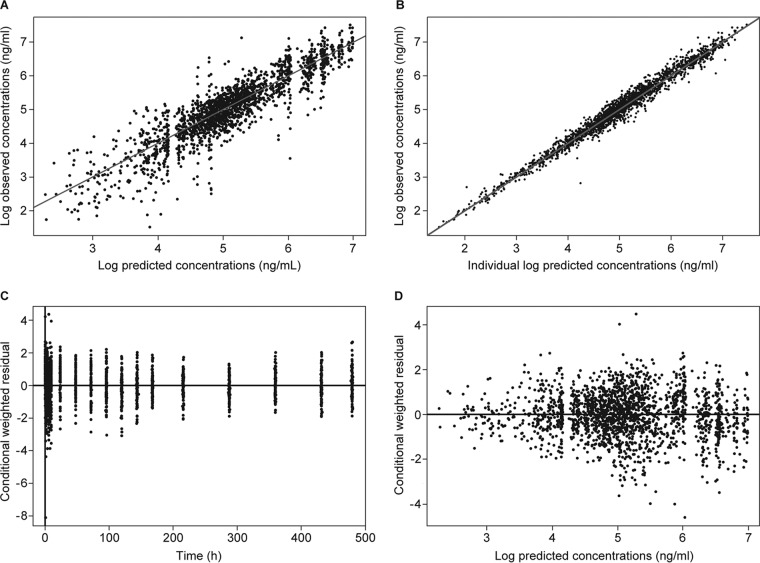
Goodness-of-fit plots for the best covariate model. (A) Log of predicted concentrations versus log of observed concentrations. (B) Log of individual predicted concentrations versus log of observed concentrations. (C) Plot of conditional weighted residual versus time. (D) Plot of conditional weighted residual versus log of predicted concentrations.

**FIG 2:**
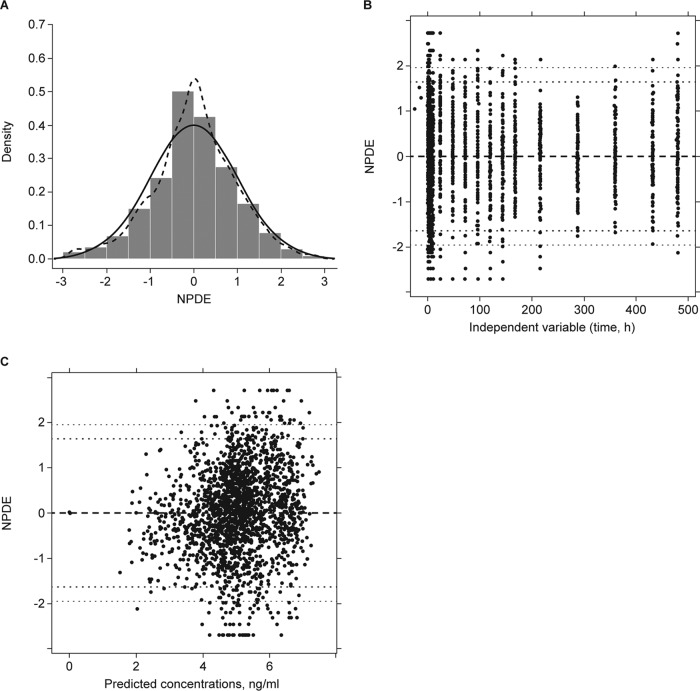
Normalized prediction distribution error (NPDE) plots. (A) Histogram of NPDE with the density of normal distribution and variance, N (0,1), with overlays of normalized distribution (solid line) and calculated distribution (dashed line). (B) Plot of NPDE versus time (dotted lines represent 90% and 95% prediction intervals). (C) Plot of NPDE versus predicted concentrations.

Following the development of the base model, covariates of interest were then added in stepwise manner using the forward-addition/backward-elimination procedure. The only covariate statistically significant for CL was the liver function index. The liver function index was also significant for Q. BMI was significant on *V*_p_. The best model with covariates was

$$\text{CL}=\theta_{1,8,9}\;\times \text{exp}(n_{j\text{CL}})$$

$$Q=\theta_{3,10,11}\;\times \text{exp}(n_{jQ})$$

$$V3=\theta_{4}\;\times [1+\theta_{12}\;\times (\text{BMI}-27.00)]$$

where θ_1,8,9_ are the typical values of CL for healthy subjects and those with mild and moderate hepatic impairment, respectively; θ_3,10,11_ are the typical Q values for healthy subjects and those with mild and moderate hepatic impairment, respectively; and n_*j*_ denotes difference between the true parameter value of individual *j* and the typical value of CL and Q predicted for the individual. The goodness-of-fit plots, as shown in Fig. 1, showed that the model was consistent with the observed data, with no systematic and no observable covariate trends remained with the full model. Plots of the NPDE demonstrated that the normality assumption was not rejected (Fig. 2A), and plots of NPDE versus time (independent variable) and predicted concentrations did not show any trend (Fig. 2B and C). Parameter estimates are shown in Table 4.

**TABLE 4:** Parameter estimates of the best covariate model^*a*^

Parameter	Value (SE)	% RSE	Bootstrap mean	Bootstrap 95 % CI	% shrinkage
θ1 (CL, mild), ml/h	1,550 (137)	9	1,556	1,287–1,841	
θ2 (V2), ml	51,400 (1,740)	3	51,337	48,010–54,688	
θ3 (Q2, mild), ml/h	38,800 (2,410)	6	38,904	34,315–43,521	
θ4 (V3), ml	41,0000 (12,000)	3	410,661	388,432–435,732	
θ5 (RA), h^−1^	0.653 (0.035)	6	0.651	0.581–0.727	
θ6 (GAM1)	4.57 (0.303)	6	4.55	3.97–5.22	
θ7 (KAMAX), h^−1^	0.86 (0.063)	7	0.86	0.743–1.003	
θ8 (CL, healthy), ml/h	2,540 (123)	5	2,542	2,050–3,160	
θ9 (CL, moderate), ml/h	1,326 (123)	9	1,350	1,050–3,100	
θ10 (Q, healthy), ml/h	33,678 (2,400)	7	33,562	27,860–40,080	
θ11 (Q, moderate), ml/h	63,554 (4,650)	7	63740	52,730–77,600	
θ12 BMI on V3	0.058 (0.007)	13	0.058	0.042–0.074	
F1	1.00 (fixed)				
Variability (%)					
CL	43.47 (0.035)	19	42.89	35.05–50.99	2
V2	21.23 (0.016)	36	21.09	13.10–27.85	34
RA	32.55 (0.025)	24	32.08	23.87–40.24	34
KAMAX	31.78 (0.043)	43	31.52	14.83–42.97	52
GAM1	38.07 (0.034)	24	37.73	28.47–47.16	37
V3	27.05 (0.011)	26	26.85	22.44–31.42	4
Q2	36.46 (0.029)	22	35.90	28.01–44.08	7
Residual error, σ^2^	17.88 (0.0029)	9	18.18	16.74–19.76	

a CL, clearance; Q, intercompartmental clearance; KAMAX, RA and GAM1, Weibull absorption parameters; BMI, body mass index; RSE, relative standard error; CI, confidence interval.

### Model validation.

The results from 500 bootstrap replicates are summarized in Table 4. The mean estimates of all parameters from the bootstrapping procedure were very similar to the parameter estimates from the best covariate model. This indicates adequacy and stability of the model and that the estimates of fixed and random effects were accurate.

### Simulation profile comparing healthy and hepatic impaired subjects.

**FIG 3:**
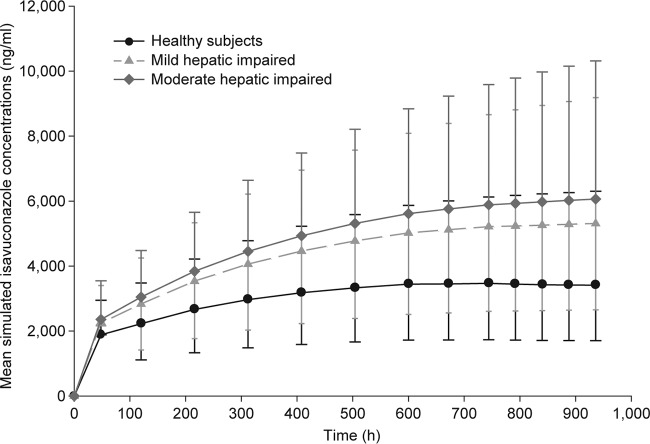
Mean simulated isavuconazole concentrations (with 95% confidence intervals) as a function of time.

Monte Carlo simulations were performed by using population estimates from the best covariate model. A total of 2,000 total isavuconazole concentration-time profiles were simulated to steady state for healthy subjects and subjects with mild and moderate hepatic impairment. The BMI values were randomly added based on values found in NHANES database (2014), with subjects between the ages of 18 and 65 years selected. The concentration-time profile was simulated using the clinical dosing regimen. Simulations showed less than a 2-fold increase in mean trough isavuconazole concentrations for subjects with both mild and moderate hepatic impairment compared with healthy subjects administered the clinical dose of isavuconazole at steady state. The mean simulated trough concentrations are presented in Fig. 3, and the summary of trough concentrations is presented in Table 5.

**TABLE 5:** Summary table of simulated trough concentrations at steady state for healthy subjects and subjects with mild and moderate hepatic impairment^*a*^

Subjects	Simulated trough concn (ng/ml) at steady state				
	Mean	SD	Median	Minimum	Maximum
Healthy	3,500	1,490	3,300	560	11,280
With mild hepatic impairment	5,300	2,000	5,067	1,189	13,700
With moderate hepatic impairment	6,068	2,202	5,840	1,548	14,330

a Values are rounded to the nearest decimal.

### Safety.

In the study including subjects with hepatic impairment caused by hepatitis B and/or C, isavuconazole was well tolerated in healthy subjects and subjects with mild or moderate liver impairment; no new safety signals for isavuconazole were observed. Treatment-emergent adverse events (TEAEs) related to laboratory test results were reported for six subjects: neutropenia (two healthy subjects, intravenous isavuconazole), decreased neutrophil count (one healthy subject, intravenous isavuconazole), and hematuria (two subjects with moderate liver impairment, one intravenous and one oral isavuconazole). One subject reported three laboratory abnormalities (increased alanine transaminase, aspartate aminotransferase, and C-reactive protein) and also experienced two serious adverse events. All TEAEs related to abnormal test results resolved, and all were considered to be remotely related or unrelated to study drug. No deaths occurred during the study, and no subjects discontinued the study due to a TEAE.

In the study including subjects with hepatic impairment caused by alcoholic cirrhosis, there were no deaths and no other serious or severe TEAEs reported during the study. No subject was permanently withdrawn from the study as a result of a TEAE. There was no evidence of a pattern of laboratory shift changes or marked laboratory values by hepatic impairment group or by dose route. One healthy subject and one subject with moderate impairment who received the oral dose of the study drug had abnormal ECG results; the abnormality in the subject with moderate impairment was reported as a TEAE of “incomplete” right bundle branch block. This abnormality was reported from 4 h through 72 h postdose but was back to normal by day 10. The healthy subject had abnormal ECG results from day 1 (2 h) through to the follow-up assessment on day 21. Single doses of isavuconazole (p.o. or i.v.) had no clinically relevant effect on vital signs or physical examination findings.

## DISCUSSION

Data regarding the PK and safety of isavuconazole in subjects with hepatic impairment due to alcoholic cirrhosis have previously been published (12). In the current analysis, a modeling procedure was conducted to combine the concentration-time data from two hepatic studies, hepatic impairment due to alcoholic cirrhosis and due to hepatitis B/C, to determine the effect of liver diseases on the PK of isavuconazole and to determine if dose adjustment is necessary. In both the above-mentioned studies, data were analyzed using noncompartmental analysis (NCA). However, insufficient sampling time points in the later part of the elimination phase for subjects with mild and moderate hepatic impairment led to a high area-under-the-curve extrapolation percentage (AUC_%ext_), which might have impacted the NCA parameters. Combining the two studies (64 subjects), 12 out of 32 subjects for the mild impaired group and 19 out of 32 subjects for the moderate impaired group had high AUC_%ext_ values (>20%) values. The AUC_inf_ and AUC_%ext_ of the above-mentioned subjects are shown in Table 6. PPK modeling was also undertaken to reduce this bias and simulate concentration-time profiles to steady state.

**TABLE 6:** AUC_inf_ and AUC_%ext_ for subjects with mild and moderate hepatic impairment^*a*^

Subject	Subjects with mild hepatic impairment (*n* = 12)		Subject	Subjects with moderate hepatic impairment (*n* = 19)	
	AUC_inf_ (h · mg/liter)	AUC_%ext_		AUC_inf_ (h · mg/liter)	AUC_%ext_
1	64	41	1	87	38
2	93	24	2	133	31
3	92	41	3	118	23
4	67	27	4	53	35
5	194	58	5	71	29
6	121	39	6	257	82
7	80	31	7	83	47
8	157	51	8	78	22
9	73	48	9	140	44
10	210	62	10	86	37
11	117	42	11	72	39
12	50	37	12	174	41
			13	113	29
			14	48	27
			15	57	33
			16	130	48
			17	34	24
			18	69	27
			19	77	30

a AUC_inf_, area under the curve to infinity; AUC_%ext_, percent extrapolation. Values are rounded to the nearest decimal.

Isavuconazole PK in subjects with hepatic impairment was best described by a two-compartment model with Weibull absorption function and first-order elimination. All parameters in the model were precisely estimated. The goodness-of-fit plots did not show any systematic bias, as all model diagnostics indicated an overall good fit of the model to the data. Internal validation for the best covariate model was performed with bootstrapping, which resulted in 95% CIs for each fixed and random effect parameter. The mean parameter values of the final model and those obtained from bootstrapping procedure were relatively similar, indicating adequacy of the developed model.

It has been established from previous studies that less than 1% of a dose of isavuconazole is excreted into the urine as unchanged isavuconazole (11). In addition, administration of rifampin has been found to reduce the exposure to isavuconazole by 97%, whereas administration of ketoconazole increases concentrations 5-fold (3). These results coupled with *in vitro* data indicate that hepatic metabolism, mediated by CYP3A4 and CYP3A5 isozymes, is primarily responsible for the elimination of isavuconazole (11).

The present analysis indicated that CL was significantly correlated with severity of liver function. Total isavuconazole CL values observed in healthy subjects were similar to values obtained previously (11). The mean CL values for subjects with mild and moderate hepatic impairment decreased approximately to 1.55 liters/h (5th and 95th percentiles: 1.3 and 1.8 liters/h) and 1.32 liters/h (5th and 95th percentiles: 1.05 and 1.35), respectively. The *V*_p_ was not significantly different in the presence of liver disease; however, it increased with increasing BMI, but that did not affect the exposure. None of the other demographics had any statistical significance on either CL or *V*_p_. The hepatic index as well as all other demographics had no statistically significant effects on any absorption parameters.

Isavuconazole given as a single dose was safe and well tolerated in subjects with mild and moderate hepatic impairment as well as in healthy subjects. The adverse event profiles for subjects with hepatic impairment were similar to those for healthy control subjects. Single doses of isavuconazole had no clinically relevant effect on vital signs or physical examination findings.

Since the two studies were conducted with a single 100-mg dose of isavuconazole rather than the recommended clinical dosage (200 mg every 8 h for days 1 and 2 and then 200 mg thereafter), isavuconazole concentrations were simulated using the clinical dosing regimen to steady state using mean parameter values obtained from a population model with covariates. From Fig. 3 and Table 5, it can be seen that mean trough concentrations of isavuconazole for healthy subjects were approximately 3,500 ng/ml, while mean concentrations for subjects with mild and moderate hepatic impairment were approximately 5,300 and 6,100 ng/ml, respectively. There was less than a 2-fold increase in mean simulated trough concentration for subjects with hepatic impairment compared with healthy subjects. The mean concentrations in subjects with moderate hepatic impairment were within the 95% CI for healthy subjects.

The PK and safety data support the notion that dose adjustment appears to be unnecessary for subjects with mild to moderate hepatic impairment, although careful monitoring of subjects is necessary for any unexpected events that might occur. Isavuconazole has not been studied for subjects with severe hepatic impairment (Child-Pugh C class) and should be used in these subjects only when the benefits outweigh the risk. Clinical monitoring for adverse reactions is recommended when treating patients with severe hepatic impairment (3).

In conclusion, a population pharmacokinetic model was developed for subjects with liver impairment. Among the parameters that could have affected exposure, only severity of liver impairment was statistically significant on CL Due to the observation that there was less than a 2-fold increase in total isavuconazole concentrations for subjects with mild to moderate hepatic impairment and no clinically relevant effect on vital signs or physical examination findings, there appears to be no need for a dose adjustment of isavuconazonium sulfate in patients that present with invasive aspergillosis and/or mucormycosis that also have mild to moderate liver impairment.
